# Evidence that spillover from Marine Protected Areas benefits the spiny lobster (*Panulirus interruptus*) fishery in southern California

**DOI:** 10.1038/s41598-021-82371-5

**Published:** 2021-01-29

**Authors:** Hunter S. Lenihan, Jordan P. Gallagher, Joseph R. Peters, Adrian C. Stier, Jennifer K. K. Hofmeister, Daniel C. Reed

**Affiliations:** 1 Bren School of Environmental Science and Management, University of California Santa Barbara, Santa Barbara, CA 93116 USA; 2 Department of Ecology, Evolution, and Marine Biology, University of California Santa Barbara, Santa Barbara, CA 93116 USA; 3 Marine Science Institute, University of California Santa Barbara, Santa Barbara, CA 93116 USA; 4 California Department of Fish and Wildlife, San Diego, CA 92123 USA

**Keywords:** Ecology, Ecology, Environmental sciences, Sustainability, Marine biology

## Abstract

Marine Protected Areas (MPAs) are designed to enhance biodiversity and ecosystem services. Some MPAs are also established to benefit fisheries through increased egg and larval production, or the spillover of mobile juveniles and adults. Whether spillover influences fishery landings depend on the population status and movement patterns of target species both inside and outside of MPAs, as well as the status of the fishery and behavior of the fleet. We tested whether an increase in the lobster population inside two newly established MPAs influenced local catch, fishing effort, and catch-per-unit-effort (CPUE) within the sustainable California spiny lobster fishery. We found greater build-up of lobsters within MPAs relative to unprotected areas, and greater increases in fishing effort and total lobster catch, but not CPUE, in fishing zones containing MPAs vs. those without MPAs. Our results show that a 35% reduction in fishing area resulting from MPA designation was compensated for by a 225% increase in total catch after 6-years, thus indicating at a local scale that the trade-off of fishing ground for no-fishing zones benefitted the fishery.

## Introduction

Marine Protected Areas (MPAs) are widely recognized as effective conservation tools for protecting marine resources within their borders^1,2^, and are used with increasing frequency in marine spatial management^3^. Research has shown repeatedly that the absence of fishing in MPAs leads to increased biomass, size, density, and diversity of protected species within well-enforced reserves^4–6^. Whether positive conservation outcomes also benefit fisheries are less certain, yet are important to assess because many MPAs are established with assurances to local fisheries that the capacity of MPAs to increase fish stocks and yield will outweigh costs associated with the loss of fishing grounds^7–9^. MPAs are thought to enhance adjacent fisheries mainly in two ways, through increased export of eggs and larvae that eventually augment populations of target species^10,11^, or through increases in biomass of animals near MPA borders that ostensibly move into fished areas and are caught as “spillover”^12–15^.

Numerous studies have reported evidence for the spillover of large, mobile taxa from MPAs into adjacent fished areas^16–19^. For example, 20 years after the implementation of a marine protected area in the Philippines, there was a tripling of fish density inside the reserve, as well as in the neighboring 200–300 m wide area just outside the reserve, a pattern attributed to spillover^20^. Patterns of density-driven spillover with potential benefits to fisheries have most often been observed for exploited species of mobile invertebrates, especially lobsters, in large part because they are intensively fished using stationary traps that can be placed at reserve borders^19,21^. Nevertheless, increased biomass and density within MPAs, and behavioral responses by fishers, such as “fishing the line”^22^, have not emerged for all reserves^23–28^. In some cases, spiny lobsters have even spilled into MPAs^29^.

Despite much attention given to documenting spillover from MPAs, relatively few studies have attempted to quantify the extent to which increases in the density and size structure of animals along the borders of MPAs influences fishing behavior, total landings, and revenue^19^. We know of two studies, both conducted on lobsters, that measured the net gain in a fishery generated by spillover^30,31^. Goñi et al.^30^ examined a heavily exploited lobster fishery in the Mediterranean Sea and found a net gain of 10% in total lobster weight caught after MPA implementation despite there being a decrease in the total number of individual lobsters caught. Moland et al.^31^ documented a 245% increase in lobster caught within reserves that was linked to an 87% increase in nearby fished areas. The net benefit to the fishery was attributed in both cases to the spillover of relatively large lobsters from MPAs. Such studies are critical for demonstrating the ability of MPAs to meet their fishery objectives because without them the implementation of MPAs risks being perceived as having unfulfilled expectations and a lack of credibility in their purported value as an effective tool for fisheries management^32^.

The long history of coastal MPAs in California (CA) provides a unique setting for studying their effects. Small coastal MPAs have existed in the state since the 1930s. Passage of the Marine Life Protection Act in 1999 led to the establishment of an additional 124 MPAs, encompassing 2197 km^2^ of protected marine habitat, distributed along 1900 km of coastline^33^. Studies conducted within CA’s MPAs have focused primarily on understanding the biological and ecological consequences of protection. For example, researchers studying a network of MPAs at the Channel Islands in southern CA documented significant positive effects of protection on a wide variety of fish and invertebrate species within and immediately outside of reserve borders^34,35^. One of the largest responses was observed in the CA spiny lobster (*Panulirus interruptus*), which increased dramatically in both biomass and size within multiple MPAs after only five years (2003–2008) of protection^21^, and exhibited spillover into adjacent fishing zones^36^. The rapid response of lobster to MPA protection was rather surprising because the lobster fishery in CA is considered relatively sustainable^37^. Whether the gain in lobster abundance in fishing zones adjacent to MPAs is sufficient to compensate for the reduction in fishable area created by the designation of MPAs has yet to be determined.

The establishment of a network of no-take zones in 2012 along the mainland coast of southern CA, where CA spiny lobster is heavily fished, provided us with a unique opportunity to study the influence of MPAs and spillover on the lobster fishery and to determine its potential benefit to the commercial lobster fishery. Prior observations of spillover of lobster from MPAs within five years of establishment at the offshore Channel Islands^33^ suggested that a similar pattern would emerge along the adjacent mainland coast, especially because the mainland fishery is more heavily fished than that at the Channel Islands^27,38^. Therefore, we tested the hypothesis that the establishment of MPAs along the mainland coast of the Santa Barbara Channel would lead to increased lobster biomass and density inside MPAs, which in turn would lead to increased landings in the region despite the reduction in fishable area caused by the establishment of the MPAs. Our approach involved analyzing: (1) fishery independent data collected by divers within and outside of MPAs, immediately before and six years after MPA establishment, to determine the effects of the MPAs on lobster density, size and biomass, and (2) fishery dependent landings data of lobster catch and fishing effort, also collected six years before and after MPA establishment, to assess the effects of the MPAs on the fishery. Our results are important to fishers, who were assured spillover would eventually enhance lobster catches, and fishery managers, who worked in collaboration with fishers to established reserves with fishery benefits.

## Results

### Fishery independent estimates of lobster abundance and size

**Figure 1:**
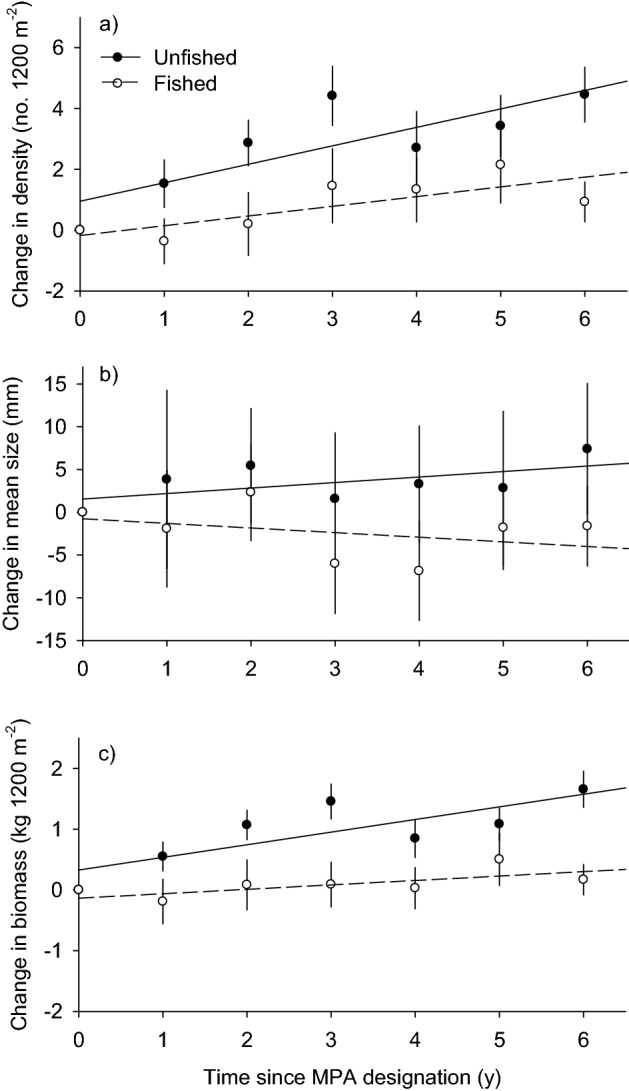
Annual changes in the: (**a**) density, (**b**) mean carapace length and (**c**) biomass of spiny lobster in fixed 1200 m^2^ plots within MPAs and fished areas off the coast of Santa Barbara since the establishment of MPAs in 2012. Values are means with 95% CI. N = 17 and 19 for unfished and fished plots respectively.

The density of lobster increased significantly over time in both fished and unfished plots, however the rate that density increased in unfished MPA plots was three times higher than that in fished plots (Fig. 1a; *F*_1.248_ = 5.53, *P* = 0.020 for protection status × time interaction). By contrast, the mean carapace length of lobster did not change significantly over time in either fished or unfished plots (Fig. 1b; *F*_1.182_ = 2.12, *P* = 0.147 for protection status × time interaction), although it’s worth noting the non-significant trends in fished and unfished plots showed opposite patterns with size increasing over time in the unfished plots and decreasing in the fished plots. Similar patterns of non-significance and opposing directional trends were observed for changes in the median carapace length of lobster (*F*_1.182_ = 2.16, *P* = 0.143, data not shown), which is more sensitive to skewed distributions.

Observed changes in lobster biomass following MPA establishment closely resembled those of lobster density (Fig. 1a vs. c). Biomass increased significantly over time following MPA establishment in both fished and unfished plots (*F*_1.131_ = 4.65, *P* = 0.033 and *F*_1.118_ = 62.45, *P* < 0.0001 for fished and unfished plots, respectively), with threefold higher increases in unfished MPA plots compared to fished plots (Fig. 1c; *F*_1.248_ = 12.14, *P* < 0.001 for protection status × time interaction).

### Fishery dependent estimates of lobster catch and fishing effort

**Figure 2:**
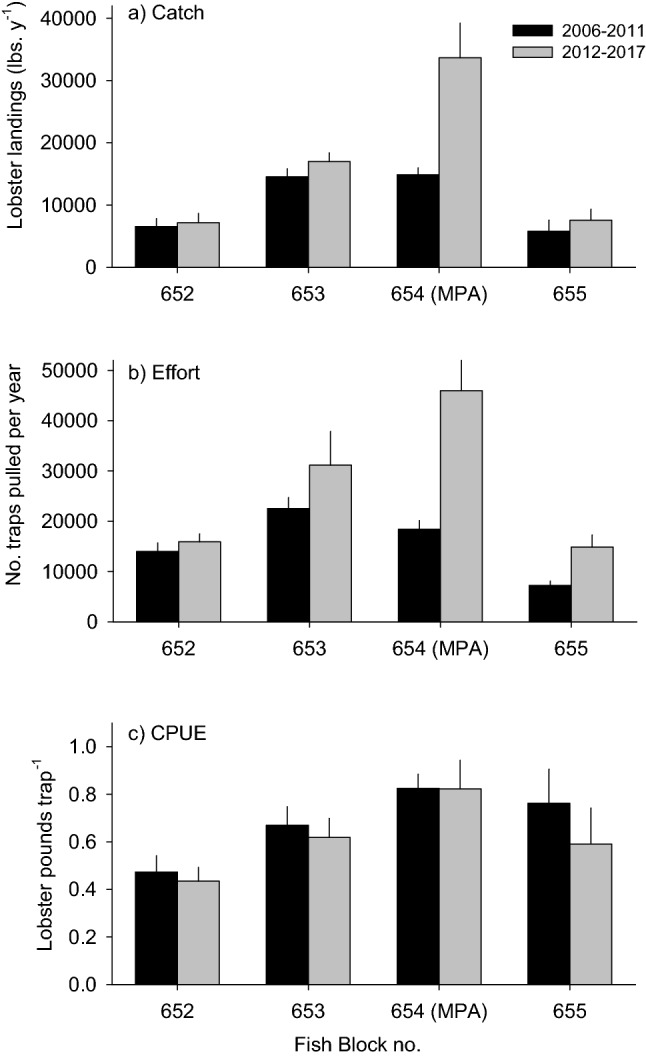
CDFW fishing block data for: (**a**) catch, (**b**) effort and (**c**) catch per unit effort (CPUE) of spiny lobster off the mainland coast of Santa Barbara. Values are means ± SE for the 6-year periods before and after the establishment of MPAs in block 654.

Analyses of commercial fishing block landing data showed that the effects of fishing block on the total annual catch of lobster differed significantly between the six-year periods before and after MPA establishment (Fig. 2a; *F*_3.40_ = 4.86, *P* = 0.006 for block × time period interaction). Average annual lobster landings more than doubled in the 6 years after MPA establishment in the block with the two MPAs (#654) despite a 35% reduction in the fishable area (Table 1). In contrast, lobster landings in the three blocks without MPAs remained relatively unchanged during the six years following MPA establishment.

**Table 1:** The fishable area of state waters within the CDFW fishing blocks off the coast of Santa Barbara before and after the establishment of MPAs in 2012.

Block number	Fishable area (ha) pre-2012	Fishable area (ha) post-2012	Percent reduction (%)
652	9589.9	9589.9	0
653	9555.3	9555.3	0
654^a^	9665.7	6257.0	35
655	9015.1	9015.1	0

^a^Denotes fishing block with MPAs.

Annual fishing effort estimated by the number of traps pulled increased throughout the study region following MPA establishment with the largest increase occurring in block 654 (Fig. 2b; *F*_3.40_ = 4.31, *P* = 0.010 for block × period interaction). CPUE differed significantly among the fishing blocks (Fig. 2c; *F*_3.40_ = 4.54, *P* = 0.008), but not between the periods before and after MPA establishment (*F*_1.40_ = 0.84, *P* = 0.850). Block 654 with the two MPAs consistently had the highest CPUE irrespective of the timing of MPA establishment. The nearly identical values of CPUE in block 654 before and after MPA establishment suggests the doubling of landings in this block in the six years following the establishment of the two MPAs resulted from a doubling of the fishing effort rather than an increase in lobster stock.

The average annual catch in the study region summed across all four fishing blocks increased by 57% in the six years following MPA establishment while the fishing effort increased by 73%. By contrast, the average annual CPUE of the region declined slightly from 0.68 (± 0.051 SE) to 0.61 (± 0.058 SE) pounds per pull.

## Discussion

Spiny lobster density and biomass increased in MPAs and fished areas in the 6 years following the establishment of the two no fishing zones. The increase in lobster abundance across the region observed in our fishery independent data may be related to a suite of factors that we did not test, including enhanced lobster recruitment related to El Nino events in 2006–2007, and 2014–2016^39^. However, the increase within the MPAs was four times greater for density, and almost twice as much for biomass than in the fished areas, indicating lobster abundance increased dramatically in the absence of fishing. Similar increases in lobster populations have been observed within established no-fishing zones at the Channel Islands^40^ and elsewhere^12,41^, but over a substantially longer period of time (15–23 years). That the change in mean size of lobsters did not vary markedly inside vs. outside of MPAs is interesting but not unexpected. Prior work comparing lobster sizes inside vs. outside MPAs in the Channel Islands has been equivocal concerning effects on mean carapace length: Iaccheci et al.^41^ found little difference for relatively long-established MPAs while Kay et al.^21^ found large positive effects of MPAs only 5 years after they were established.

Cessation of fishing appears to have been the primary mechanism driving the relatively large difference in lobster density and biomass inside vs. outside of the MPAs. Nevertheless, other ecological mechanisms may have also played a part, including spatial variation in lobster habitat and recruitment, and differences in food availability. Prior work in the Santa Barbara Channel found that the intensity of lobster spillover from MPAs across multiple reserves was driven mainly by cessation of fishing, and to a lesser degree by the spatial arrangement of the caves, cracks, and crevices where lobsters gather to shelter from their predators^36^. While we did not quantify the amount and quality of lobster habitat in this study, we have no reason to believe that those habitat metrics changed over the course of the study. Of course, habitat availability is a key factor in spillover because as lobster density increases inside reserves preferred shelters become crowded leading to the movement of lobsters to less-crowded habitats outside of the MPAs^36^. The role of recruitment in generating the patterns that we report remains uncertain. Peters et al.^39^ found evidence that recruitment was lower in the MPAs than in fished areas in 2014 but observed the opposite pattern in 2015. They found no difference in recruitment inside vs. outside of MPAs in 2012–2013 and 2016–2018.

We have no direct evidence that lobster populations increased inside the MPAs due to greater food availability. An indirect ecological effect of ceasing to fish lobsters on temperate latitude rocky reefs is an increase in the abundance of macroalgae—specifically giant kelp in the SB Channel- due to the decline of sea urchins, which graze on kelp and are preyed upon by lobsters^42^. In turn, increased kelp abundance can lead to greater abundances of many species of invertebrates, some of which are lobster prey^43^. We do not have the data to test this idea but other work in our region indicates that lobster prey species tend to decline instead of increase inside MPAs, apparently due to increased lobster forging^44^. In summary, indirect ecological effects related to the cessation of fishing may have contributed to the large increase in lobster abundance in the MPAs, but do not adequately explain the dramatic differences we report between fished and unfished areas.

Fishery-dependent catch data showed that lobster landings in the fishing block 654 with MPAs increased by ~ 225% after the reserves were implemented compared with an average increase of 19% in the three blocks without MPAs. Importantly, the large increase in catch in fishing block 654 occurred despite a 35% decrease in fishable area, and resulted from a 250% increase in effort, as catch efficiency (i.e., CPUE) remained relatively constant. The pattern of increased effort and constant CPUE implies there was a substantial increase in the abundance of legal sized lobsters in the fishable area of fishing block 654 after the MPAs were established, which is consistent with the results of the diver surveys. By contrast, the CPUE in the three fishing blocks without MPAs declined by an average of 13% in the six years after the MPAs were established. We predict that this pattern was driven mainly by the most efficient fishers who shifted their effort to fish close to the MPA borders. To our knowledge, this is the largest increase in lobster catch associated with MPA spillover ever recorded.

The most plausible mechanism leading to the increase in lobster abundance and catch in the fishing block with MPAs was the movement of legal-sized lobsters from within the reserve boundaries to areas outside where they were caught. Such movement from the Naples SMCA and Campus Point SMCA may be facilitated by extensive rocky reefs, the preferred habitat of spiny lobster, that extend uninterrupted from within the MPA’s boundaries to areas outside where fishing occurs. Such habitat corridors are well-known to facilitate lobster movement^45^. Prior work indicates a lack of such habitat corridors reduces, or even impedes, lobster movement and spillover^21,38^. Prior work in the northern Channel Islands also suggests that substantial increases in population abundance within reserves takes place within 5 years of reserve establishment, and that increases in abundance are positively associated with greater movement of legal-sized lobsters^21^. Considered together, this is strong circumstantial evidence that substantial increases in lobster abundance within multiple MPAs over a six-year period increased the local commercial catch through spillover.

Interestingly, fishing effort also increased in nearby fishing blocks without MPAs (albeit at a much smaller rate) between 2012 and 2017, indicating that there was an overall increase in fishing effort in the region following the establishment of MPAs. This is consistent with results from visual surveys of deployed lobster traps (which are marked by surface floats), which implied that the increase in fishing pressure was concentrated at the borders of the two MPAs in fishing block 654. For example, the average number of traps counted along the eastern border of the Campus Point SMCA during the second week of the lobster fishing season nearly doubled from 46 ± 16 traps per hectare in 2009–2012 to 89 ± 14 traps per hectare in 2014–2019 (Authors, *unpublished data*). The higher fishing effort and catch coupled with a relatively constant CPUE in block 654 suggests fishers were attracted to the MPAs because of enhanced catch from spillover. This is born out in interviews with fishers who acknowledged that they purposefully fish the borders of the Naples and Campus Point SMCAs to maximize their catch (H. Lenihan, *personal communications*).

Theory suggests that MPAs benefit fisheries the most when they are poorly managed and overfished prior to reserve implementation^31,46,47^. Empirical evidence so far supports this prediction^19,30,48,49^. For example, Halpern et al.^50^ used statistical analyses of 17 MPA-fishery systems to suggest that spillover can replenish a highly depleted fishery that targets a mobile species when: (1) the gradient of decay in population abundance across the MPA’s border is steep, (2) catch is greater than the population growth rate outside the reserve, and (3) there are multiple reserves subsidizing the fishery, such as in a reserve network. A synthesis of field studies by Goñi et al.^19^ also reported patterns consistent with MPAs replenishing heavily exploited fisheries in which the abundance of target species in fished areas was far below that in no-fishing zones^51^. In addition to heavy fishing pressure, other important elements stimulating high catch outside of MPAs appear to be habitat characteristics that promote the movement of animals across MPAs borders^26,36^, good fishing habitat in close vicinity (e.g., < 800 m) to MPA borders^36,50^, and a behavioral response by the fishery to fish near the borders.

Our results provide one of the few examples that link increased abundance of a target species within reserves to increased catch within the fishery, albeit at a local scale (see review by Goñi et al.^18^). In his review Hilborn^52^ reiterated the growing consensus, gleaned from models and empirical work^19,31,50,53–55^, that MPAs increase the catch outside of no-fishing zones when fishing pressure is very high and stocks are seriously overexploited. By contrast, we have shown in this study that MPAs can increase catch in a relatively sustainable fishery. Certainly, the CA spiny lobster fishery is heavily exploited but remains sustainable, due in large part to management efforts designed to control fishing effort, and oceanic connectivity that delivers larvae to southern CA from less-heavily exploited fishing grounds in Baja California, Mexico^37,56^. An important general lesson re-emerges from our results: close collaboration between fishers, scientists, policy makers, and fishery managers enhances marine ecosystem management^10,57,58^. California’s MPA-fishery system is an integrated, well-enforced network of no-fishing zones designed to protect productive subtidal rocky reefs that are essential habitat to populations of many target species, including CA spiny lobster. The lobster fishers helped to design the CA MPA network, and the State dedicates substantial funding to effective enforcement. Our results indicate that by responding to the no-fishing zones by fishing near MPA borders, the fishery is experiencing a net benefit of no-fishing zones at a local scale. The next step is to test whether southern California’s MPA network is enhancing total catch across the entire CA spiny lobster fishery.

## Methods

### Spiny lobster fishery in southern California

The U.S. commercial fishery for spiny lobster in CA extends from Point Conception south to the U.S.-Mexico border and involves fishers using relatively small boats to deploy baited wire box-like traps set on the bottom in shallow (typically < 20 m) reef habitats. The fishing season is from October to March with approximately 80% of the annual catch landed within the first half of the season. It is assumed that the majority of lobsters landed by the fishery reached legal size (83 mm carapace length) during the previous year, but this is as yet been unconfirmed. Recreational fishing in the study region off Santa Barbara has been historically low relative to other regions in southern CA and accounts for a relatively small fraction of the total catch^59^.

### Fishery independent estimates of lobster abundance and size

**Figure 3:**
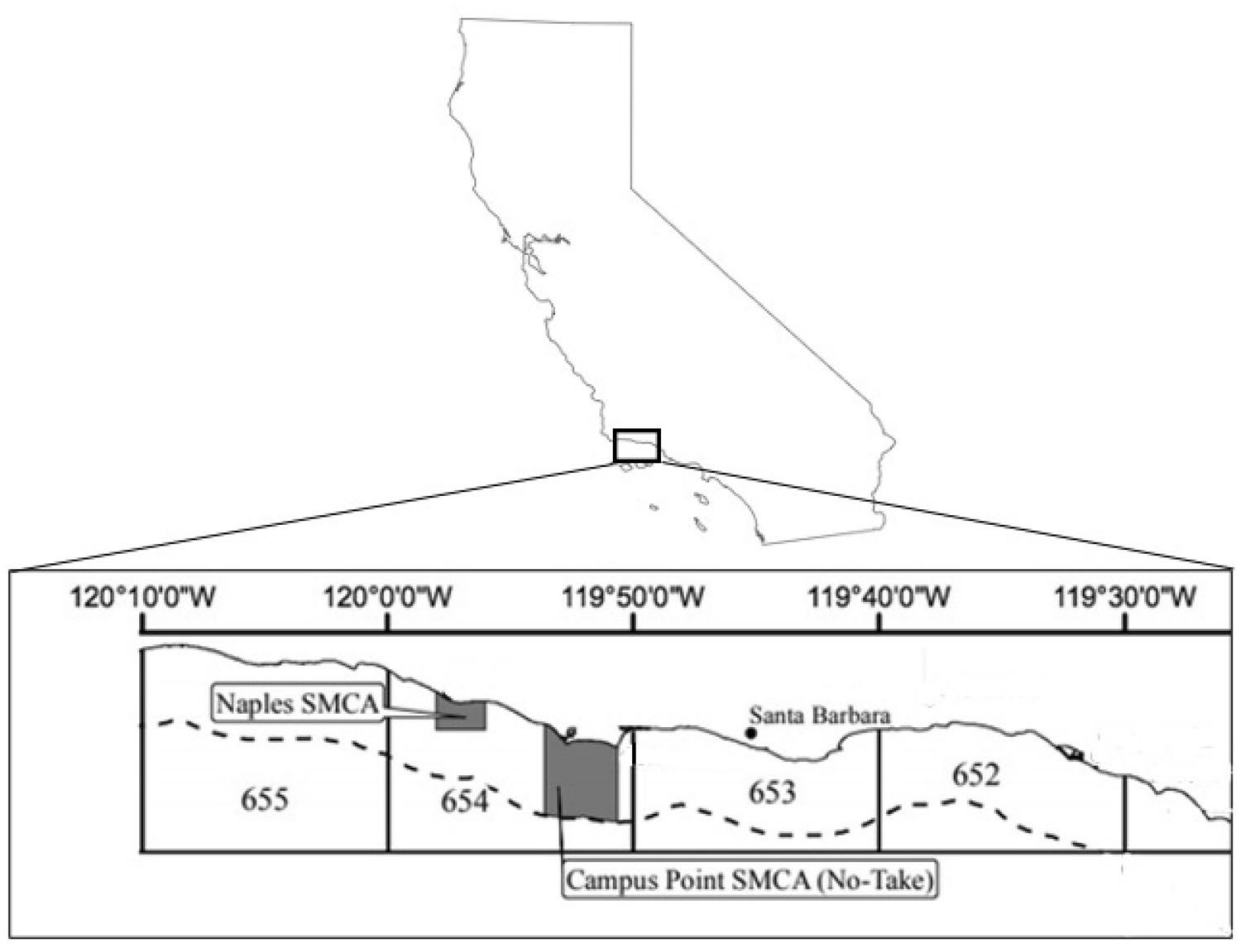
Map of CDFW designated fishing blocks off the mainland coast of Santa Barbara, CA USA. The dashed line marks the 3 nautical mile offshore state boundary of the fishing blocks. Grey polygons show the locations of the two MPAs at Campus Point and Naples Reef established in 2012. (For inset map of MPAs, see California Department of Fish and Wildlife (2021). https://nrm.dfg.ca.gov/FileHandler.ashx?DocumentID=135356&inline, p. 4.).

Underwater surveys of lobster abundance and size were conducted using scuba by the Santa Barbara Coastal Long Term Ecological Research program in reef habitat at five sites spanning 75 km of the mainland coast of the Santa Barbara Channel: Arroyo Quemado (AQUE; 120.07°W, 34.28°N), Naples (NAPL; 119.57°W, 34.25°N), Isla Vista (IVEE; 119.51°W, 34.24°N), Mohawk (MOHK; 119.43°W, 34.23°N), and Carpinteria (CARP; 119.32°W, 34.23°N). Two of these sites, NAPL, and IVEE, are located within MPAs established in 2012 (i.e., Naples SMCA and Campus Point SMCA, respectively), while the other three sites (AQUE, MOHK, and CARP) have always been open to fishing (Fig. 3). At each site divers recorded the number and visually estimated the size (i.e., carapace length) of lobster in 1200 m^2^ plots (n = 2 to 8 plots per site depending on the available reef habitat). Using handheld lights divers thoroughly searched the benthos, including areas of dense vegetation, crevices, ledges, and other structures used by lobster to shelter, to obtain a census of lobster in each plot (n = 36 plots total). Surveys were conducted annually from 2012 to 2018 during daylight hours in late August to mid-September prior to the start of the lobster fishing season to control for possible confounding effects of habitat type, storms, season, and time of day on lobster abundance (sensu Iacchei et al.^41^). Details of the sampling methodology and data can be found in Reed^60^.

We classified plots as fished (n = 17) or unfished (n = 19) depending on whether or not they were located in an MPA. The effects of protection status (fish vs. unfished) and time since MPA establishment on the absolute change in lobster density, mean carapace size, and biomass since MPA established were determined in separate ANCOVAs in which protection status was considered a fixed factor and time since MPA establishment a covariate. The mass of each lobster (wet g) was calculated as 0.001 × L^2.914^ where L is the carapace length in mm^61^. Lobster biomass was calculated as the summed mass of all lobster observed in a plot. Lobster survey data were not collected prior to 2012 when MPAs were established.

### Fishery dependent landings data

CA fisheries are managed by the CA Department of Fish and Wildlife (CDFW), who has divided the entire coastline into rectangular fishing blocks (~ 140 km^2^), from which commercial lobster fishers, and other fisheries, are required to record and log all their catch (https://nrm.dfg.ca.gov/FileHandler.ashx?DocumentID=67449&inline). Annual fishing block data of commercial landings (wet kg caught) and fishing effort (number of traps pulled) of spiny lobster in the study region were obtained from CDFW for the six fishing seasons prior to MPA establishment (2006–2011) and the six seasons after MPA establishment (2012–2017). We defined a fishing season by the year in which it started (e.g., the 2012 season extended from October 2012 through March 2013). Fishing block data are based on landings weighed at the dock by the processor, who records the data on a “fish ticket” that is submitted to the CDFW. Fishermen are required to assign their landings to a specific fishing block and report the fishing effort (i.e., number of traps pulled) allocated to their catch. Catch-per-unit-effort (CPUE) is defined by CDFW as the number of legal lobsters per trap pull. Herein, we define CPUE as the weight of lobsters pulled per traps pulled.

The study region included four commercial fishing blocks that extend along the mainland coast of the Santa Barbara Channel (Fig. 3). The two MPAs (Naples SMCA and Campus Point SMCA) are located in a single block (#654) and their designation in 2012 constituted a 35% reduction in the fishable area in this block (Table 1). The CA Marine Life Protection Act had no effect on the amount of fishable area in the other three fish blocks in the study region (# 652, 653, 655).

The effects of fishing block and period (i.e., before vs. after MPA establishment) on annual catch, annual effort, and annual catch-per-unit-effort (CPUE) were evaluated in separate fixed factor ANOVAs. Landings and effort data were log transformed to meet the assumptions of normality and homoscedasticity. All statistical analyses were conducted in SPSS-SYSTAT software.

### Ethical approval

All methods were carried out in accordance with University of California and National Science Foundation guidelines and regulations. All experimental protocols were approved by the University of California, Santa Barbara and the California Department of Fish and Wildlife.
